# Dating of polyhalite: a difficult ^40^Ar/^39^Ar dating tool of diagenetic to very low-grade metamorphic processes

**DOI:** 10.1007/s00531-022-02219-9

**Published:** 2022-07-07

**Authors:** C. Leitner, F. Neubauer, J. Genser, M. Bernroider

**Affiliations:** Department of Geography and Geology, University of Salzburg, Hellbrunner Strasse 34, 5020 Salzburg, Austria

**Keywords:** Polyhalite, Ar–Ar age dating, Microfabrics, Fluids, Haselgebirge Formation

## Abstract

Halite already deforms at surface temperatures. A valuable universal dating tool to constrain the timing of sedimentary, diagenetic, or deformational structures is still missing. The evaporite mineral polyhalite can be dated by the ^40^Ar/^39^Ar method. On the example of the extremely deformed halite deposits of the Eastern Alps, polyhalite was tested to date early diagenetic stages of the deposits. The sedimentological investigation of the present study indicates that some of the macrostructures of polyhalite had a syn-depositional origin during the late Permian. It is supposed that polyhalite originated during reflux of brines. All samples selected for age dating represent characteristic microfabric types of euhedral to subhedral polyhalite crystals. Intact macro- and non-recrystallized looking microstructures of polyhalite can be expected to give plateau ages. However, nearly all measurements produced overdispersed data that do not define an age. The oldest age steps thus represent only minimum ages. A closer look revealed grain boundary migration, subgrain rotation recrystallization, twinning, and fluid-supported grain size increase. These recovery processes obscured the original ages and/or reflect the origin of new polyhalite in place of the original individuals. Based on these microstructures, the age data are supposed to reflect the circulation of aqueous fluids. Just extremely careful separation of individual crystals or in situ age dating under the microscope will be successful in dating polyhalite. Nevertheless, polyhalite can potentially serve to date deformational events of halite deposits due to its easy recrystallization property.

**Supplementary Information:**

The online version contains supplementary material available at 10.1007/s00531-022-02219-9.

## Introduction

Major evaporite successions are generally mobilized during post-depositional processes and form complex assemblages in compressional and extensional structures, e.g., in diapirs, raft tectonics in epicontinental settings, or collisional mountain belts (Warren 2016). These structures are often of high economic importance, e.g., for salt mining, hydrocarbon deposits and repository for nuclear wastes. However, there is still no tool available to allow dating of the structural evolution, besides stratigraphic relationships to country rocks. Potassium containing minerals are commonly used to date geological events by the ^40^Ar/^39^Ar method. Polyhalite [K_2_Ca_2_Mg(SO_4_)_4_·2H_2_O] is a K-bearing mineral of evaporite deposits. It forms under sedimentary or diagenetic conditions, which makes this mineral interesting as a potential geochronometer for sedimentary, diagenetic or low-temperature tectonic processes.

Synthetic polyhalite becomes unstable by dehydration between 250 and 350 °C under laboratory conditions (Freyer and Voigt 2003; Wollmann et al. 2008; Wollmann 2010). A natural polyhalite from the Permian Salado Formation, USA, started to dehydrate already at 507°K = 233 °C. Using in situ synchrotron X-ray diffraction, the polyhalite decomposed into vapor, anhydrite and two langbeinite-type phases with different Ca/Mg ratios (Guo and Xu 2017; Xu et al. 2017). The closure temperature has been attempted to determine only recently in the year 2021. It was assessed by regressing five steps of degassing between ca. 400 and 600 °C. This temperature range is however far beyond the dehydration temperature of polyhalite (ca. 230 to 350 °C). After dehydration, likely melting of langbeinite solid solutions occurred at 1143.7 K (≈ 870 °C) (Xu et al. 2017). Therefore, not the closure temperature of polyhalite was determined. The closure temperature of the langbeinite-type phases was calculated between 250 and 280 °C for a cooling rate of 10 °C/Ma (Richards et al. 2021).

Polyhalite age dating by Rb/Sr and K/Ar methods was tried out in the past, but most ages were difficult to interpret within their geological frame (Pilot and Blank 1967; Brookins et al. 1980; Brookins 1986; Halas et al. 1996; Wójtowicz et al. 2003). Onstott et al. (1995a) used ^40^Ar/^39^Ar dating of polyhalite, however, only total gas ages were mentioned in this abstract, and no full data table was attached. Analytically advanced dating, including ^40^Ar/^39^Ar step heating, has been performed only on the chemically related mineral langbeinite [K_2_Mg_2_(SO_4_)_3_]. Some samples yielded plateau ages, and discordant age spectra were interpreted as mixtures of two or more generations (Lippolt and Oesterle 1977; Lippolt et al. 1993; Léost et al. 2001; Renne et al. 2001). Recently, alunite [KAl_3_(SO_4_)_2_(OH)_6_] was successfully dated (Ren and Vasconcelos 2019). Both sulfates were stable to temperatures high enough to determine their closure temperature. Some polyhalite from the Eastern Alps was already dated by the authors, however with similar difficulties to interpret as in the other regions (Leitner et al. 2013, 2014, 2020).

**Fig. 1:**
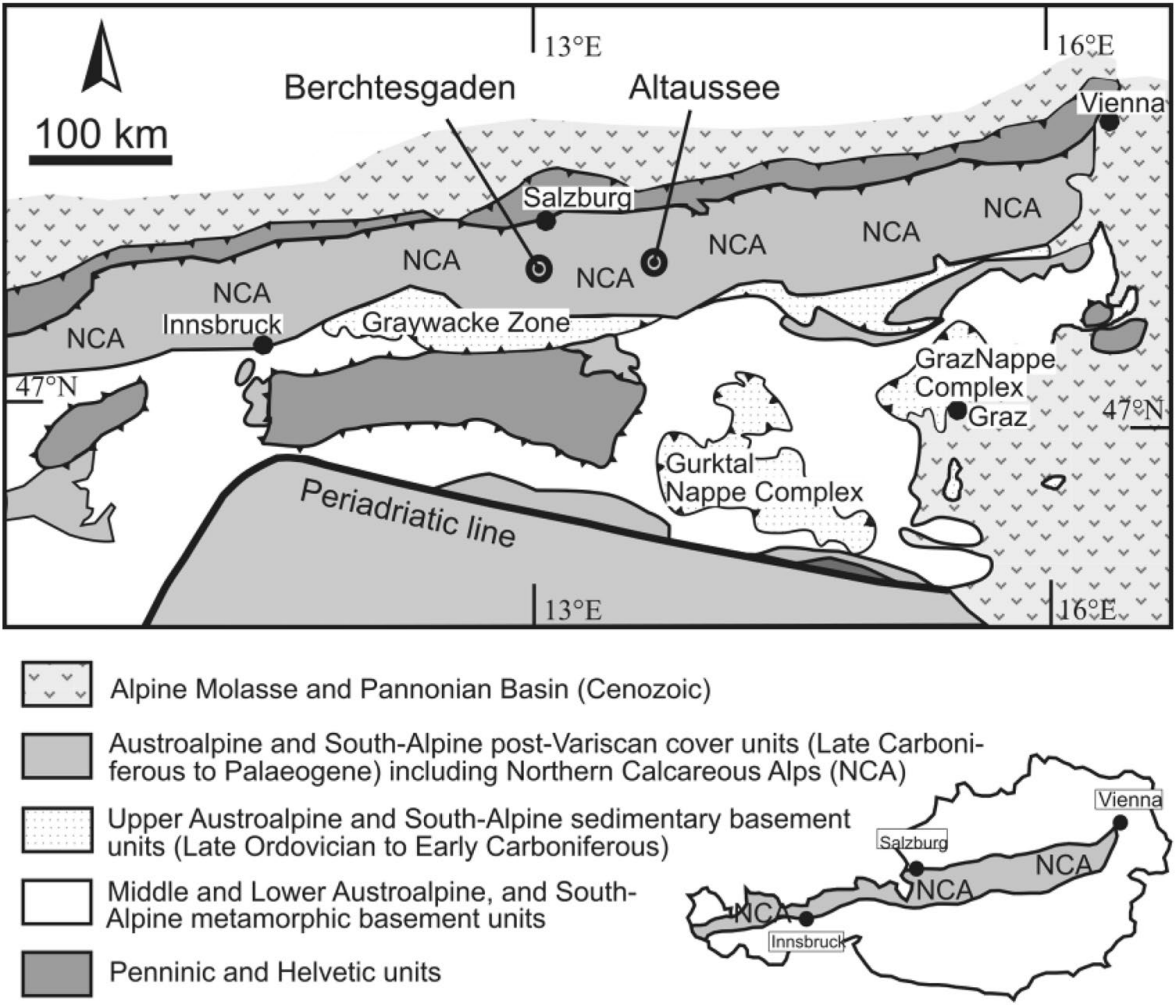
Tectonic map of the Northern Calcareous Alps as part of the Eastern Alps, location of the Bad Dürrnberg-Berchtesgaden and Altaussee salt deposits

All measurements of this study were conducted on samples from the evaporitic Alpine Haselgebirge mélange of the Northern Calcareous Alps, which is the type region of polyhalite (Stromeyer 1818; Schlatti et al. 1970; Bindi 2005) (Fig. 1). The sedimentological investigation of the late Permian Haselgebirge indicates that some of the macrostructures of polyhalite have a syn-depositional origin. Such structures have never been described before but put the Alpine deposits in a row with other polyhalite deposits worldwide (Warren 2018 and references therein).

In this study, only polyhalite of intact syn-sedimentary macrostructures and non-recrystallized looking microfabrics of polyhalite were selected and the results of this age dating are presented in this paper. However, it turned out that what looked undisturbed at a cursory glance is, in fact, no longer pristine when examined in sufficient detail. The nomenclature of representative polyhalite fabrics was further improved relative to Leitner et al. (2014). The samples were measured multiply to test the reproducibility of age dating and the robustness of the data on polyhalite. The new data should be seen in complement to earlier results presented by the authors.

## Geological setting

The Northern Calcareous Alps represent a cover fold-and-thrust belt (Fig. 1) with uppermost Pennsylvanian to Eocene sedimentary successions. In their central and eastern sectors, evaporites of the Haselgebirge Formation were dated to late Permian (~  250 Ma, Piller et al. 2004) by paleontological and geochemical methods (Klaus 1965; Spötl and Pak 1996). The evaporites were deposited in an aborted rift setting (Spötl 1989). Stratigraphically younger, up to 3.5 km of shallow water carbonates were deposited on the passive margin facing towards the Neotethyan ocean (Mandl 2000). During convergent tectonics, the stratigraphic level of these evaporites acted as a décollement level. Evaporite-decorated thrusts are known in all structural levels of the Northern Calcareous Alps since a long time (e.g., Weber 1958, 1997a; Schauberger et al. 1976; Tollmann 1976; Wessely 2006; Lobitzer 2011; Mandl et al. 2012; Granado et al. 2019; Fernandez et al. 2020). The thrusting and propagation direction of thrusting was from east/southeast to west/northwest (Linzer et al. 1995; Mandl 2000; Schorn et al. 2013a). The main thrusting event between late Jurassic and early Cretaceous was associated with high-grade diagenetic conditions (~ 200 °C) in the southern and central parts of the Northern Calcareous Alps (Bojar et al. 2016 and references therein). Finally, the NCA fold-and-thrust belt was transported over the stable European lithosphere during late Eocene and superimposed by Oligocene to Miocene strike-slip faulting.

## Materials and methods

### Locations and material

Samples were taken from two salt bodies. The salt body of Altaussee (UTM 33 T 405316 5278325) has a vertical thickness of > 800 m. The salt body of Bad Dürrnberg-Berchtesgaden (UTM 33 T 351091 5278007) is at least 1000 m thick and crops out c. 60 km west of the other deposit. The dominant rock type in both salt bodies is a protocataclasite of halite and mudstone (Leitner et al. 2011). Samples were selected from the Altaussee (ALT), Bad Dürrnberg (DÜ) and Berchtesgaden (BGD) salt mines (Fig. 1).

### Electron microprobe analysis

Measurements were performed on a JEOL electron microprobe (JXA-8600), equipped with a wave-length dispersive system. An acceleration voltage of 15 kV and a low sample current of 20 nA were applied to prevent decomposition of polyhalite under the electron beam. Additionally, the spot was defocused to a diameter of 15 µm and after measurement of sulfur, the sample was moved one beam diameter to start measurement of potassium and calcium. Sulfur, potassium and calcium were all measured with the same analyzing crystal. Synthetic and natural mineral standards were used to analyze the emitted wave lengths of the sample and to quantify their amount. Standard ZAF correction calculation revealed the composition in oxide weight percent. The calculation method after Love/Scott1 revealed the formula units of polyhalite.

### ^40^Ar/^39^Ar technique

Polyhalite samples were manually reduced to small pieces with a hammer. They were washed with distilled water and dried with isopropanol to free them from dust and Cl-ions of halite. Chlorine produces Ar isotopes during irradiation, which may tamper the proportion of Ar isotopes from polyhalite. Grains of 200–250 µm size were selected under the microscope. Enough grains of each sample were packed into aluminum-foil and put into quartz vials.

Details of the analytical ^40^Ar/^39^Ar technique is described in Leitner et al. (2014) and Cao et al. (2017). Irradiation was conducted for 16 h in the Magyar Tudományos Akadémia (MTA) Központi Fizakai Kutato Intézet (KFKI) reactor (Debrecen, Hungary). No Cd shielding was applied. Flux-monitors were placed between the samples for calculation of the J-values. The distance between adjacent flux-monitors was c. 5 mm. Corrections for interfering isotopes were the same as described earlier: Correction factors were calculated from 45 analyses of co-irradiated Ca-glass samples and 70 analyses of K-glass samples, and are: ^36^Ar/^37^Ar_(Ca)_ = 0.000225, ^37^Ar/^39^Ar_(Ca)_ = 0.000614, ^38^Ar/^39^Ar_(K)_ = 0.0117, and ^40^Ar/^39^Ar_(K)_ = 0.0266. Variation in the flux of neutrons were monitored with the DRA1 sanidine monitor for which a ^40^Ar/^39^Ar plateau age of 25.26 ± 0.05 Ma has been reported (van Hinsbergen et al. 2008).

^40^Ar/^39^Ar analyses were carried out at the Department of Geography and Geology at the University of Salzburg. The equipment used was the same as described earlier: ^40^Ar/^39^Ar analyses are carried out using a ultrahigh vacuum Ar-extraction line equipped with a combined MERCHANTEK™ UV/IR laser system, and a VG-ISOTECH™ VG-3600 noble gas mass spectrometer. Stepwise heating analyses of samples are performed using a defocused (~ 1.5 mm diameter) 25 W CO_2_-IR laser operating in Tem_00_ mode at wavelengths between 10.57 and 10.63 µm. The laser is controlled from a PC, and the position of the laser on the sample is monitored on the computer screen via a video camera in the optical axis of the laser beam through a double-vacuum window on the sample chamber. Gas clean-up is performed using one hot and one cold Zr-Al SAES™ getter. Gas admittance and pumping of the mass spectrometer and the Ar-extraction line are computer controlled using pneumatic valves. The VG-3600 is an 18 cm radius 60° extended geometry sector field mass analyzer instrument, equipped with a bright Nier-type source operated at 4.5 kV. Measurements are performed on an axial electron multiplier in static mode, peak-jumping and stability of the magnet is controlled by a Hall-probe. For each increment the intensities of ^36^Ar, ^37^Ar, ^38^Ar, ^39^Ar, and ^40^Ar are measured, the baseline readings on mass 35.5 are automatically subtracted. Intensities of the peaks are back-extrapolated over 16 measured intensities to the time of gas admittance either by a straight line or a monotonically decreasing exponential, depending on intensity and type of pattern of the evolving gas.

Inspection of intensities was applied regarding background, system blanks, interfering isotopes, and post-irradiation decay of ^37^Ar. Calculations of isotope ratios, errors, ages and plateau ages followed suggestions of McDougall and Harrison (1999), Scaillet (2000), Steiger and Jäger (1977), Ludwig (2012), and Schaen et al. (2020).

## Results

### Polyhalite types—macrostructures and microfabrics

Polyhalite occurs in a wide variety of structures. In the following, polyhalite macrotypes are distinguished, which were already recognized in Leitner et al. (2013). Thereby, the nomenclature of polyhalite fabrics is based on four different macrofabric types. In the present paper, the further differentiation of types is based on a subdivision of the fourth type, bedded polyhalite. The microfabric subdivision uses crystal shapes, i.e., (1) blocky, (2) large lath-shaped and (3) tiny lath-shaped.

All polyhalites tested by electron microprobe were chemically pure. The content of subordinate cations was low and similar, with oxide percentages below 0.3 percent (Mn < Na < Sr < Fe) (Supplementary Information 1).

### Halite-anhydrite-polyhalite nodules

**Fig. 2:**
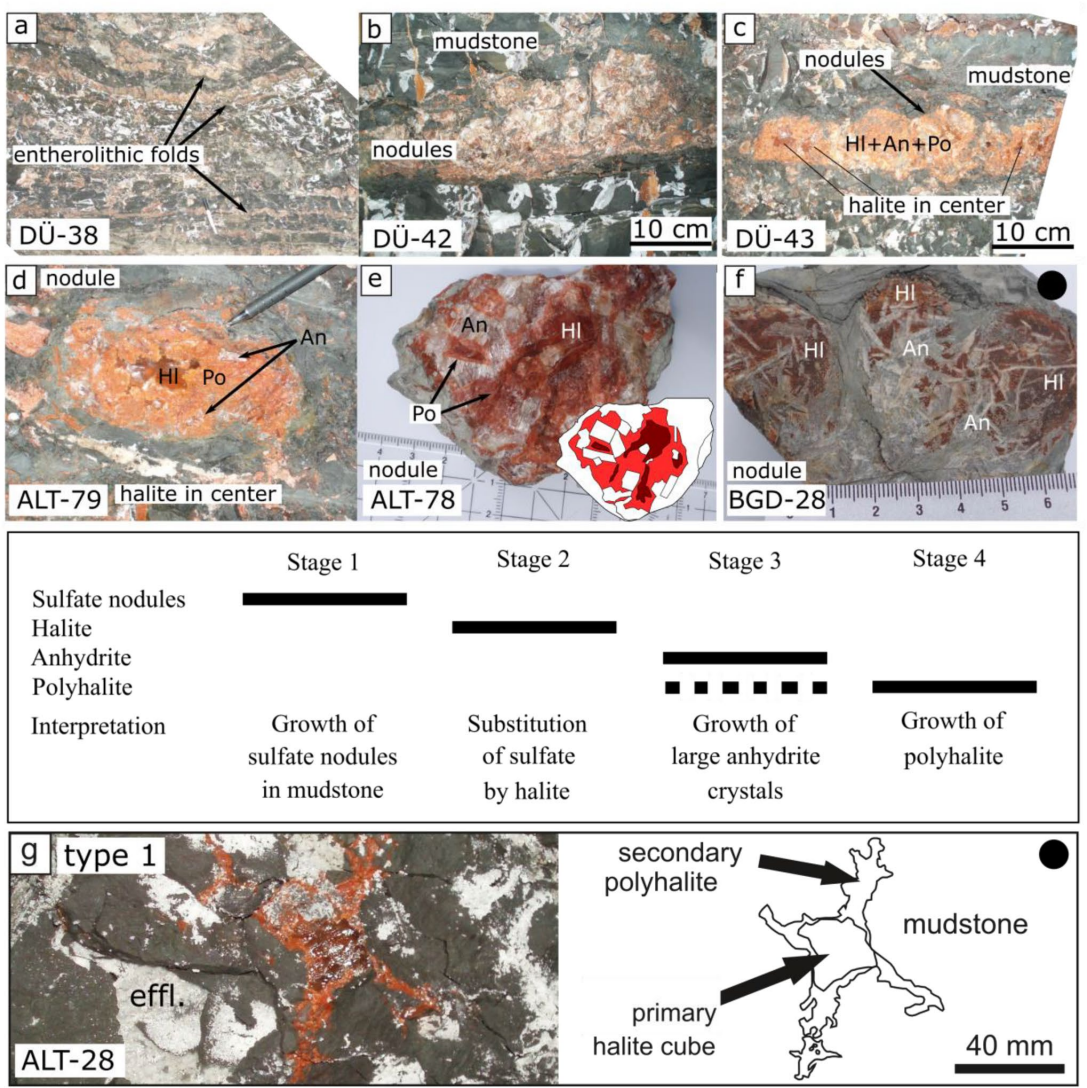
Halite-anhydrite-polyhalite nodules and polyhalite type 1 (polyhalite substituting halite). **a**–**c** Enterolithic folds and nodular structures. **d**–**f** Some of the nodules consist of halite + anhydrite + polyhalite. Chart gives relative ages of minerals within nodules: Sulfate nodules most likely formed in a sabkha environment. Sulfate was replaced by halite first (!), and later by anhydrite and polyhalite. **g** Halite in displacive halite crystals was replaced by polyhalite. Images from Leitner et al. (2013) are marked with a black dot. Abbreviations: *Hl* halite, *An* anhydrite, *Po* polyhalite, *effl.* efflorescent halite

Some enterolithic folded layers and nodules in mudstone consist of halite, anhydrite and polyhalite (Fig. 2a–b). Halite is always present in the center of such nodules (Fig. 2c–d). Large anhydrite crystals of 0.5–2 cm are arranged along the rim of the nodule. The anhydrite crystals can be prismatic or lath-shaped (Fig. 2e–f). Polyhalite is located between the large anhydrite crystals and halite. Polyhalite forms seams of fibers around the anhydrite crystals. Larger polyhalite sometimes also forms euhedral lenticular crystals in contact to halite. Nodules of this type were found in the Altaussee, Bad Dürrnberg, and Berchtesgaden mines (Fig. 2a–f).

The nodules display the typical form of sabkha nodules, which form by evaporation of a mudflat. It is supposed that sulfate was replaced by halite first (see “Discussion”). Anhydrite and polyhalite substituted halite within the nodule shape. Anhydrite and polyhalite replaced halite in the same relative age relation as in displacive halite crystals (Fig. 2g).

### Samples used for age dating

#### Type 1, polyhalite replacing halite

Polyhalite partly replaced halite of a displacive halite crystal in mudstone in sample ALT-28 (Fig. 2g). The hoppered halite crystal displays an edge length of c. 1.2 cm. Substitution was from outside towards the center by forming a polyhalite margin. The polyhalite fibers are oriented normal to the surface of the crystal. The average grain size of polyhalite is 1–2 mm, however single fibers reach 4–5 mm in length.

#### Type 2, polyhalite veins in mudstone

**Fig. 3:**
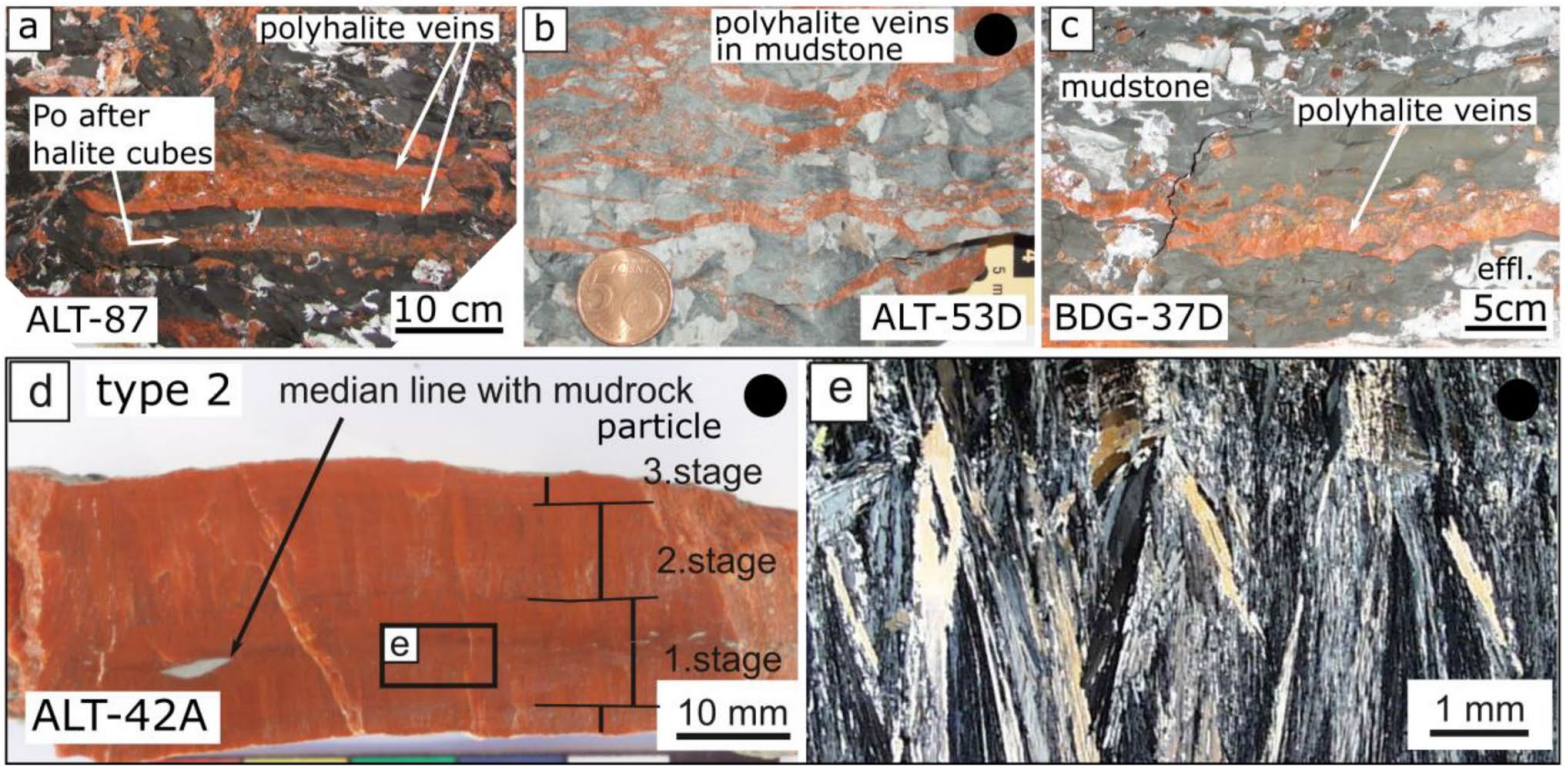
Polyhalite type 2 (polyhalite veins). **a**–**c** Antitaxial polyhalite veins subparallel to the layering in mudstone. **d** Fan-shaped fibers normal to layering, several growth stages. Serrated fibers with undulatory extinction. Images from Leitner et al. (2014) are marked with a black dot. Crossed polarizers. Abbreviation: *Po* polyhalite

Polyhalite veins in mudstone are typically arranged subparallel to the sedimentary layering. Location ALT-87 shows polyhalite types 1 and 2 next to each other: Polyhalite substituted displacive halite crystals, but additionally, veins of polyhalite developed above and below this layer (Fig. 3a). Sample ALT-53D displays several connected polyhalite veins. Most of them are oriented subparallel to the layering of the mudstone. The vertically oriented fibers are several millimeters in length (Fig. 3b). Sample BGD-37D was taken from a fibrous polyhalite vein from the Berchtesgaden mine. The vein in mudstone is c. 3 cm in thickness. The polyhalite fibers are up to 2.5 cm large (Fig. 3c). Sample ALT-42A is a polyhalite vein of around 2 cm in thickness. It shows at least three stages of antitaxial fibrous polyhalite growth. Mudstone particles exist in the median line from the initial crack. In the inner zone, the fibrous grains are 2–3 mm in size. The outer parts show fan-shaped fibers, up to 7 mm in length. The growth direction was from inside towards the wall rock. The fibers show undulatory extinction, a serrated habit, and grain boundary migration. Small anhydrites (0.2 mm) are distributed randomly over the fibers. They include polyhalite and are themselves partly corroded (Fig. 3d).

#### Type 3, polyhalite porphyroblasts in anhydrite

**Fig. 4:**
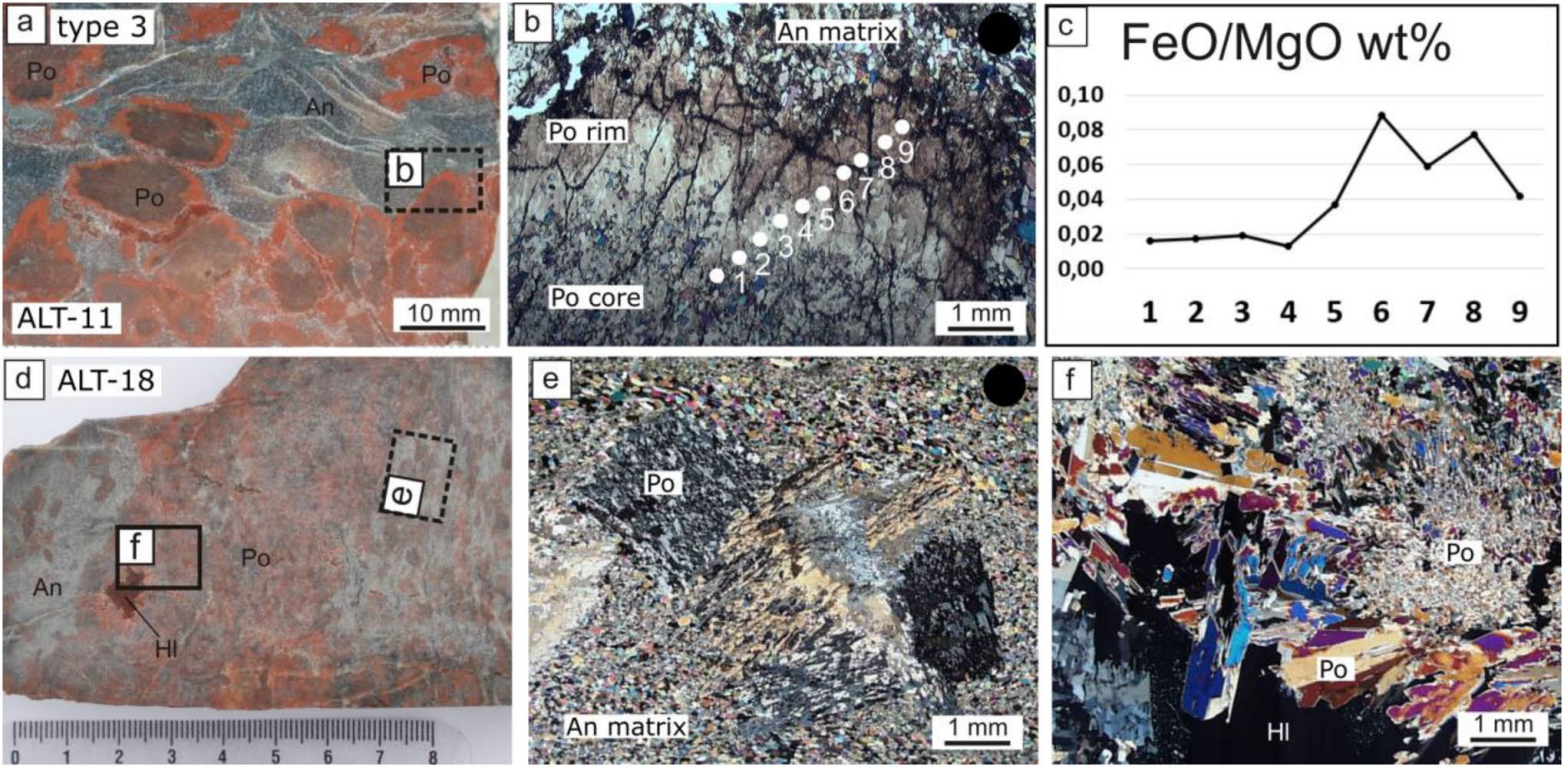
Polyhalite type 3 (polyhalite porphyroblasts in anhydrite). **a**–**c** Patchy structure from undulatory extinction. The iron content varies significantly from core to rim. **d**–**e**. Decomposed looking porphyroblasts, interpreted as subgrain rotation recrystallization. **f** Grain size increase towards halite. Images from Leitner et al. (2014, 2020) are marked with a black dot. Crossed polarizers. Dashed line indicates approximate position. Abbreviations: *An* anhydrite, *Hl* halite, *Po* polyhalite

Porphyroblastic polyhalite grains grew within an anhydrite matrix. The polyhalite grains of sample ALT-11 are up to 8–10 mm in diameter. The central part is dark red, whereas the rim is bright red in color. Core and rim are part of the same crystal under the microscope. Polyhalite displays a patchy structure from undulatory extinction (Fig. 4a–b). The surrounding groundmass consists of anhydrite with an average size of 0.1–0.2 mm. The equigranular, polygonal fabric shows a slightly preferred orientation of anhydrite. The polyhalite grains contain numerous anhydrite inclusions. The bright red margins relate to an increased Fe content (Fig. 4c).

Sample ALT-18 consists of anhydrite with polyhalite porphyroblasts (Fig. 4d). Under the microscope polyhalite prisms are locally circumfluent by elongate anhydrite crystals. The polyhalite prisms are blocky to elongate. The internal structure of the polyhalite porphyroblasts looks decomposed but is of similar birefringence color. These structures are interpreted as subgrain rotation recrystallization (Fig. 4e). A halite patch is surrounded by light red polyhalite. The large halite patch is interpreted to be a former displacive halite crystal, which persisted during the transformation of anhydrite into polyhalite. Euhedral polyhalite crystals are located at the contact to halite. Tiny-grained patches of probably decomposed earlier large polyhalite crystals surround them (Fig. 4f).

#### Type 4, bedded polyhalite

**Fig. 5:**
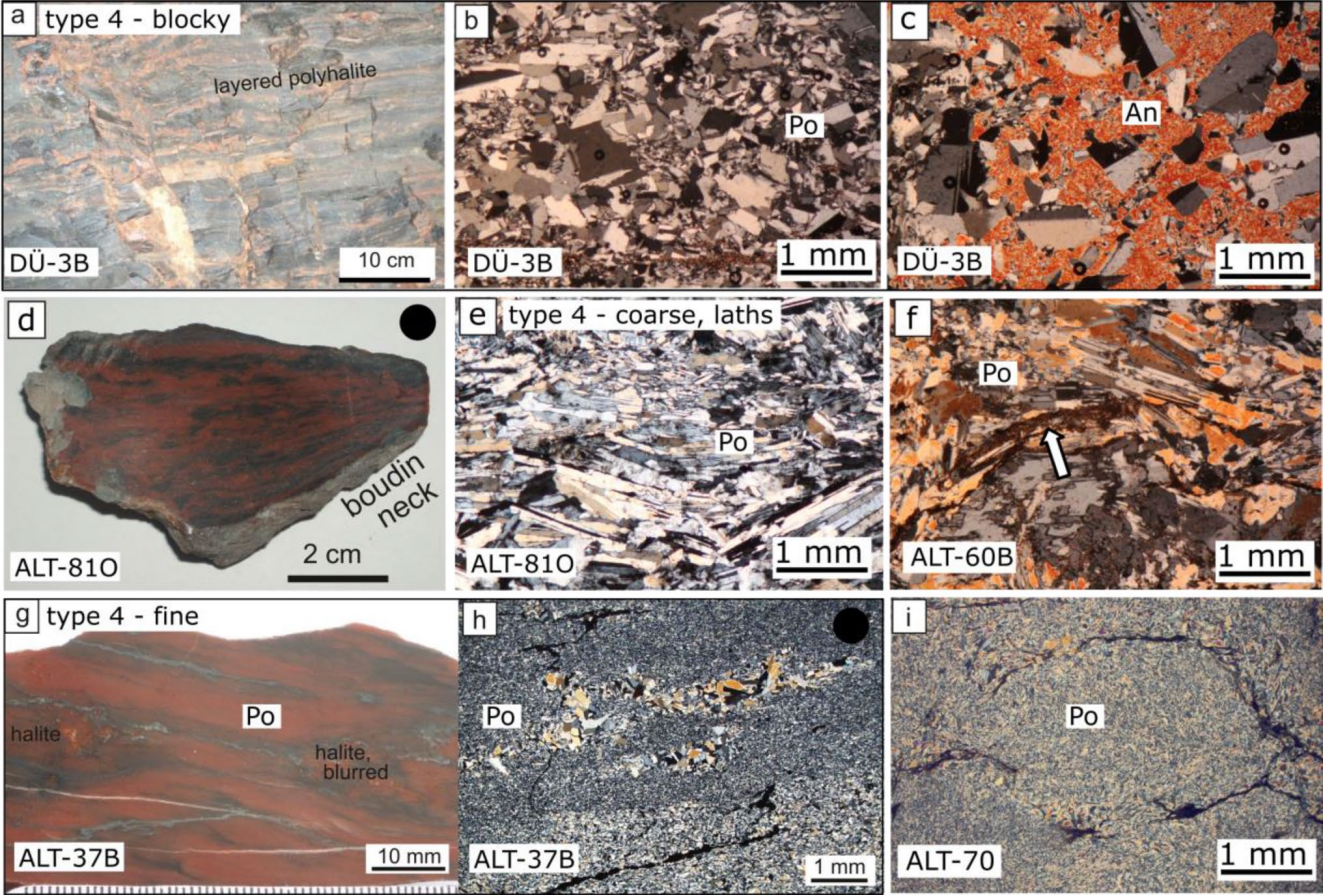
Polyhalite type 4 (bedded polyhalite). **a**–**c** Blocky polyhalite with twins, locally overgrown in a poikilotopic manner by large anhydrite crystals. **d**–**f** Macroscopically foliated polyhalite, which displays large lath-shaped crystals under the microscope, interpreted as recrystallization and grain size increase. **g**–**i** Tiny lath-shaped polyhalite. **g**–**h** Schlieren on macroscale, foliation, and domains of different grain size under the microscope. **i** Nodular structures outlined by mudstone; felted, but non-aligned tiny lath-shaped polyhalite. Abbreviations: *An* anhydrite, *Po* polyhalite

The most common type of polyhalite is bedded polyhalite. Bands of varying red and gray massive polyhalite mark the sedimentary layering or foliated layering, respectively. A basis to separate the many different microfabrics under the microscope is the shape and grain size of the polyhalite crystals: (1) blocky-shaped polyhalite crystals, (2) large lath-shaped polyhalite crystals, and (3) tiny lath-shaped polyhalite crystals. The large lath-shaped polyhalite crystals are usually oriented subparallel to the bedding. The tiny lath-shaped polyhalite crystals sometimes form a felted microstructure (Fig. 5). Microfabrics of bedded polyhalite can show a combination of these polyhalite crystal types and show additional structures. Deformational structures comprise shear zones, broken large anhydrite, strain shadows, or the alignment of grains. For more detailed information about deformational structures see Schorn et al. (2013b) and Leitner et al. (2014, Fig. 3).

1. Microfabrics of blocky polyhalite: Sample DÜ-3B was taken from a bedded polyhalite. The color of the polyhalite was unusually reddish gray. Only under the microscope, a blocky shape and a grain size of 0.1–0.5 mm was recognized. Towards mudstone the grain size becomes smaller. The grains exhibit the common symmetric polyhalite twinning, but multiple twinning and even chessboard-like twinning structures are present, too. The grains show a slightly shape preferred orientation parallel to the sedimentary layering. Large anhydrite crystals of several centimeters in size grew over the polyhalite and included the polyhalite grains in a poikilotopic manner (Fig. 5a–c).
2. Microfabrics of large, lath-shaped polyhalite: The macroscale sample ALT-81O represents a boudin neck, which formed during foliation of competent polyhalite layers. The blurry red and black bands show thinning and convergence at one end of the sample. Interestingly, the rock looks widely intact under the microscope (Fig. 5d). The laths are oriented subparallel to each other and subparallel to the layering. The laths display twinning parallel to the long axis of the crystal. The grain size ranges from 1 to 50 µm. Sample ALT-81O was dated with the Ar–Ar method, displaying only age steps between 140 and 190 Ma that do not define an age (Leitner et al. 2020). Sample ALT-60B displays a coarse grain size with laths up to 1 mm in size (Fig. 5e). Nevertheless, also small grain sizes of 100 µm are present in the upper left corner. Twinning is parallel to the long axis of the grains. The grains show a preferred E-W orientation on the photograph. The clay particles were squeezed by the growing polyhalite. (Fig. 5f, arrow).
3. Microfabrics of small to tiny lath-shaped polyhalite: Sample ALT-37B consists of the typical massive red shining polyhalite (Fig. 5g). It shows macroscopically schlieren, which contain some mudstone and blurry halite. Parallel layers of different grain size between 0.05 and 0.1 mm are visible under the microscope. The crystals possess a slightly shape preferred orientation and display lobate grain boundaries. In certain domains, coarser polyhalite of 0.1–0.3 mm size exhibits no preferred orientation, but straight grain boundaries. Black lines of mudstone are not aligned straight to the orientation of the foliation layers (Fig. 5h). Sample ALT-70 displays a similar tiny grain size. However different, the microstructure is felted, and not aligned. A nodular structure is recognizable by arrangement of the clay particles. The nodular structure suggests a substitution of former sulfate nodules (Fig. 5i).

### ^40^Ar/^39^Ar age dating

**Fig. 6:**
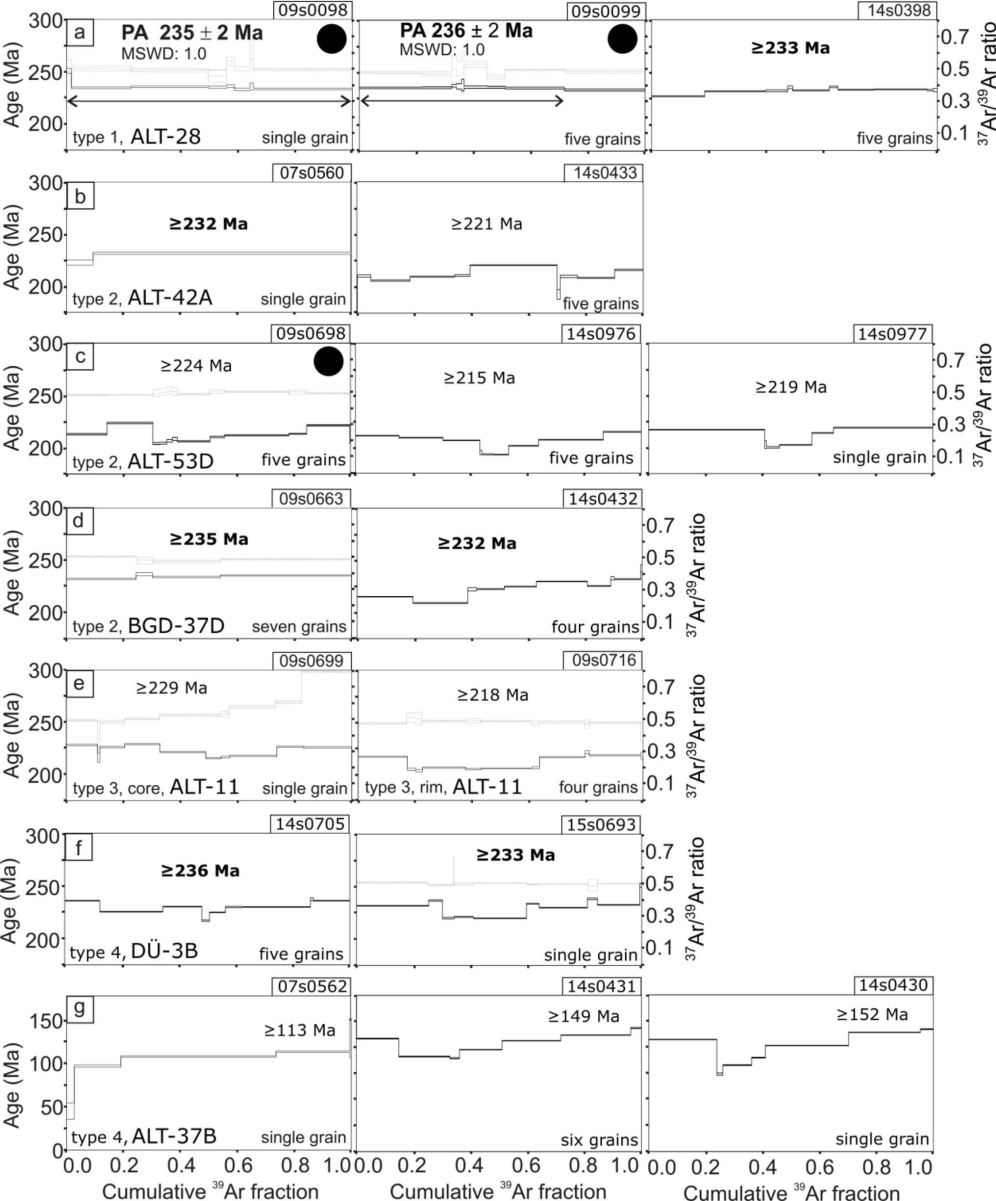
Age dating results of ^40^Ar/^39^Ar step-heating experiments. Measurements taken from earlier publications are marked with a black dot: ALT-28, measurements 09s0098 and 09s0099 (Leitner et al. 2013), ALT-53D, measurement 09s0698 (Leitner et al. 2014). Laser-power increase from left to right. All oldest ages are minimum ages, see discussion about the interpretation of ages. For samples with correction of post-irradiation decay of ^37^Ar, the ^37^Ar/^39^Ar ratio is plotted. Abbreviations: *PA* plateau age (Ludwig 2012), *MSWD* mean square of weighted deviates

Fragments of individual crystals of the 0.20–0.25 mm fraction were separated from samples ALT-28, ALT-42A, ALT-53D, BDG-37D, ALT-11 and DÜ-3B under the binocular. However, no confirmation was possible that the single grains were really individual crystals. The single grain of ALT-37B was a polycrystalline aggregate due to the tiny grain size of this sample. During multiple grain measurements, 4–7 grains were measured together. All reported errors in the text correspond to the 2σ-level (95.5% confidence level). Full results are given in Supplementary Information 2 and are graphically shown in Fig. 6. The patterns of three already published measurements in Leitner et al. (2013, 2014) are shown for comparison.

Degassing of argon occurred within a narrow range of laser power. Due to the unknown Ar retention behavior uneven degassing occurred, and between 3 and 13 steps were measured. When sufficient steps were measured, the ^37^Ar/^39^Ar (Ca/K) = 0.49 ratio was relatively constant for those samples, where correction for the post-irradiation decay of ^37^Ar was applied (09s0098, 09s0099, 09s0698, 09s0663, 09s0699, 09s0716, 15s0693) (Fig. 6, Supplemental Information 2). The ^37^Ar/^39^Ar (Ca/K) ratio of all measured steps was plotted against age and indicates some variation in the chemical composition of the polyhalite grains (see “Discussion”). For the other measurements, the time between irradiation and measurement was more than ten times the half-life of ^37^Ar and therefore the ^37^Ar/^39^Ar (Ca/K) ratio was much lower than the typical value, between 0.10 and 0.01 (14s0398, 07s0560, 14s0433, 14s0976, 14s0977, 14s0432, 14s0705, 07s0562, 14s0431 and 14s0430). Because ^37^Ar was nearly the same as background, corrections for Ca-derived ^36^Ar and ^39^Ar could not be applied. The error on the age based on the post-irradiation decay of ^37^Ar will be not more than 0.6 Ma and, therefore, lies within the error of all presented ages. ^38^Ar/^39^Ar-ratios like that of K-rich silicate glass (= 0.0117) indicated that no significant portions of Cl-ions were in the measured sample.

Sample ALT-28 (Fig. 6a), type 1, from the Altaussee mine yielded a plateau age of 235 ± 2 Ma for measurement 09s0098 (98.3 percent ^39^Ar released; Leitner et al. 2013), and measurement 09s0099 yielded a plateau age of 236 ± 2 Ma (71.8 percent ^39^Ar released; Leitner et al. 2013). Measurement 14s0398 yielded an age of around 233 Ma for the last ca. 60 percent ^39^Ar released. However, because of an MSWD of 3.9, this age was not considered a plateau age. The age is interpreted to represent a minimum age (“ ≥ 233 Ma”). Disturbed age patterns are considered to relate to overprint (see “Discussion”), and therefore, all oldest age steps are interpreted to represent only minimum ages. This is indicated by the sign “ ≥ ” on the plots in Fig. 6 for all samples discussed in the following.

Three samples of the vein type 2 were measured. Only three steps were measured for sample ALT-42A (Fig. 6b) from a single grain during measurement 07s0560. Ninety percent of ^39^Ar were released in one step with an age of 232 ± 2 Ma. The Ca/K ratio as seen in ^37^Ar/^39^Ar-ratios of the core is higher than the typical value of 0.49, which possibly relates to occasional small anhydrite crystals as observed in thin section. Five grains were measured in nine steps in experiment 14s0433. They gave a heavily disturbed age pattern. The oldest age of 221 ± 1 Ma was measured, when 31.5% of ^39^Ar were released. Similar disturbed age patters were measured for ALT-53D (Fig. 6c). Measurement 09s0698 revealed an oldest age step of 224 ± 2 Ma (five grains; Leitner et al. 2014), measurement 14s0976 an oldest age step of 215 ± 1 Ma (five grains), and measurement 14s0977 an oldest age step of 219 ± 1 Ma (single grain). The youngest ages were measured during each of the three measurements, when between 30 and 50% of ^39^Ar were released. Measurement 09s0663 of seven grains of sample BGB-37D (Fig. 6d) from the Berchtesgaden mine yielded a slightly disturbed argon release pattern with a maximum age of 235 ± 1 Ma (46% released). An aliquot under laboratory number 14s0432 showed a heavily disturbed age pattern, when four grains were measured. Thereby, the last step gave an oldest age step of 232 ± 1 Ma (9.3% of ^39^Ar released).

Fragments of the core and the rim of sample ALT-11 (Fig. 6e), type 3, from the Altaussee mine were measured separately. The core yielded a somewhat disturbed argon release pattern. The Ca/K ratio is elevated, which probably relates to the numerous inclusions of anhydrite. The pieces from the rim also yielded a disturbed release pattern. However, the ages of core and rim overlap and should thus be seen as of the same age within uncertainty. The oldest age of the core was a step at 229 ± 1 Ma.

Bedded polyhalite, type 4, was measured in two samples. The coarse-grained sample DÜ-3B (Fig. 6f), from the Bad Dürrnberg mine yielded a somewhat disturbed Ar release pattern during measurement 14s0705 of five grains. The first age step (12.3% of ^39^Ar released) and the last one (12.7% of ^39^Ar released) gave the same oldest age of 236 ± 1 Ma. Measurement 15s0693 of a single grain showed a similar release pattern. The first step (25.3% of ^39^Ar released) and the last significant step (14.9% of ^39^Ar released) gave an oldest age of 232 ± 1 Ma.

In contrast, the fine-grained polyhalite sample ALT-37B (Fig. 6g) gave much younger ages. The measurement 07s0562 of a single grain yielded a staircase pattern. After an initial age step of 44 ± 19 Ma, three large steps dominate, with 113 ± 2 Ma as the last significant step. As sample ALT-37B was proved to be pure polyhalite by EDX, the error on the age will be not more than 0.6 Ma. The aliquots of the sample resulted in heavily disturbed age patterns in measurements 14s0431 (six grains) and 14s0430 (single grain). Both measurements started with an age step like the last age step. At 20–40% of all ^39^Ar released, the youngest ages of roughly around ~ 100 Ma were measured.

## Discussion

Macroscopically primary and non-deformed structures of polyhalite were identified in the mines and under the microscope. Euhedral to subhedral polyhalite can be expected to give plateau ages in the best case. In particular, fibers, porphyroblasts and blocky shapes of polyhalite crystals looked intact and not deformed. Age dating results of these structures are discussed in the present paper. It turned out that still much work will have to be done to distinguish primary grains from recrystallized grains or parts of grains and separate them for dating.

### Primary macroscale structures

Some macrostructures indicate a syn-sedimentary origin of the Alpine polyhalite. Enterolithic folds and nodules in mudstone consist of halite, anhydrite and polyhalite. Enterolithic folds and nodules are usually typical shapes of gypsum and anhydrite, which crystallize in a sabkha environment. The sulfate nodules were pseudomorphically replaced by halite. Halite replacing sulfate has been described as a substitution during the reflux of brine through deposited evaporites (e.g., Lowenstein 1988; Hovorka 1992). In a second step, the large anhydrite crystals and polyhalite replaced the halite in the nodules. Also, during this second step, brines of different chemistry migrated through the deposit and changed the mineral composition (Fig. 2).

Polyhalite is commonly known to form from reflux brines. Polyhalite was observed to form after gypsum in recent deposits in Baja California (Holser 1966; Pierre 1983). Polyhalite formed diagenetically early in the Messinian Mediterranean salt deposit of Sicily (Lugli 1999) and in the Badenian Carpathian salt deposits (Hryniv et al. 2007; Warren 2018). Polyhalite from Polish Permian Zechstein deposits was explained by reflux brines (Peryt et al. 1998), and so was also the origin of polyhalite from the British Permian Zechstein deposit, which contains the world´s largest occurrence of polyhalite (Kemp et al. 2016). The most detailed description of polyhalite origin was given for the Permian Salado Formation in Texas, USA. Typical depositional cycles start with marine sulfates, are followed by halite, and finally end by continental muddy halite. All three parts of this typical succession contain polyhalite. A polyhalitization during each cycle was interpreted (Lowenstein, 1988). There are strong similarities of the Permian Haselgebirge Formation to the Permian Salado Formation. Both contain assemblages of mudstone, displacive halite and polyhalite. The substitution of halite by polyhalite in the Haselgebirge salt, for instance in displacive halite crystals, indicates polyhalite crystallization by reflux brines like in the other deposits.

Another major indication for an early growth of polyhalite is the development of antitaxial veins parallel to the sedimentary layering. Vertical fibers in subhorizontal veins were described in terms of crystallization power for various minerals (Hilgers and Urai 2005). The vertical fibers must lift the overburden for growth, which seems to be only possible, when the overburden is only some tens to hundreds of meters at maximum. From a sedimentological point of view, polyhalite originated syn-depositionally.

### Disturbed age patterns

Vertical fibers of antitaxial veins survived the halite foliation events because they were protected from strain mostly by the surrounding mudstone. However, a closer look showed undulatory extinction of the fibers, grain boundary migration between the fan-shaped fibers, and overgrowth by small anhydrites.

The prismatic porphyroblastic polyhalite crystals in anhydrite look primary. They originated during migration of potassium and magnesium bearing fluids through anhydrite. A closer look under the microscope revealed a patchy structure and a pattern of similar extinction orientation, which was interpreted as subgrain rotation recrystallization.

The other samples tested and discussed here, were fabrics of euhedral and subhedral polyhalite of bedded polyhalite. The best examples of non-recrystallized looking grains are large polyhalite crystals subparallel to the layering in DÜ-3B, ALT-81, and ALT-37B. They contradict the orientation of the lowest stress component indicated by the vertical fibers in the veins. On the other hand, on the macroscale the rocks clearly show signs of deformation, such as schlieren, blurry halite patches or boudins. The orientation subparallel to the pseudo-sedimentary layering represents the foliation. The grain size increased, and straight grain boundaries developed by fluid-supported recovery processes.

**Fig. 7:**
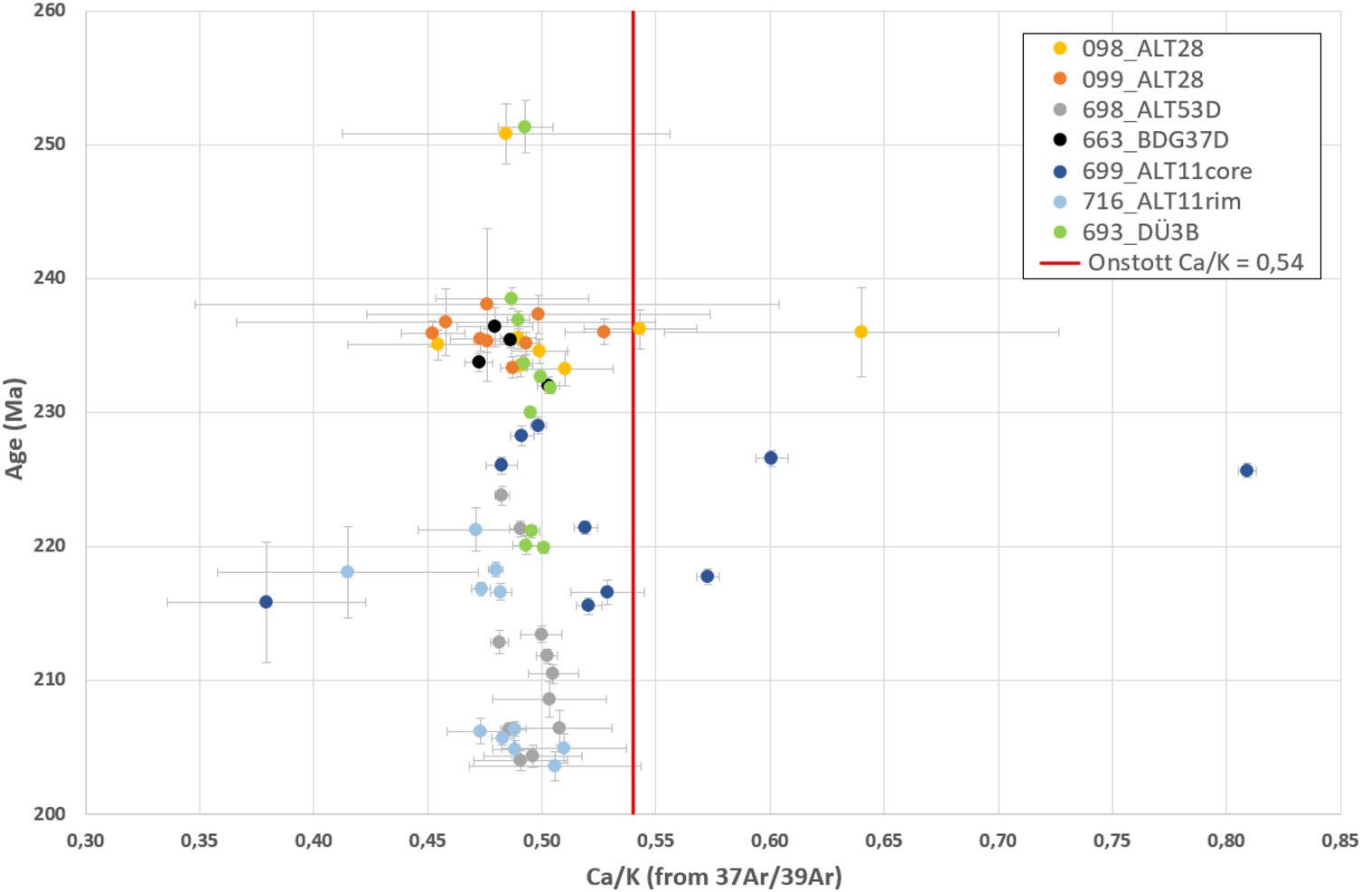
Age plotted against ^37^Ar/^39^Ar. All measured ratios are significantly lower than the theoretical value form Onstott et al. (1995b)

The ^37^Ar/^39^Ar ratio indicates chemical homogeneity or chemical variations of a material. Onstott et al. (1995b) derived the theoretical value 0.54 from particle flux of ^39^ K(n,p)^39^Ar and ^40^Ca(n,α)^37^Ar, when irradiated in a reactor, i.e., for Ca/K = 1. From the seven samples of this study (09s0098, 09s0099, 09s0698, 09s0663, 09s0699, 09s0716, 15s0693), which were corrected for post-irradiation decay of ^37^Ar, a mean value of 0.49 was calculated from the median values of the samples (Fig. 7). The Ca/K ratio is lower than expected for nearly all measurements. Is Ca stoichiometrically reduced relative to K? The EMPA data of this study seem to contradict this, because the Ca/K lies between 1.00 and 1.21, which is larger than expected for all 29 measurements (Supplementary Information 1). A possible explanation could be fractionation: While the ratio of the atomic masses of ^40^Ca and ^39^K from microprobe trends to be Ca/K > 1, the ratio of atomic masses of ^37^Ar and ^39^Ar from mass spectrometer trends to be Ca/K < 1. However, at the present state of knowledge, there is simply too little knowledge about polyhalite to explain this difference, and more baseline studies are needed.

When the ratio ^37^Ar/^39^Ar of the measured steps of the corrected samples is plotted against age (Fig. 7), some interpretations can be made. The core and the rim of ALT-11, as well as the aliquots of ALT-28, which gave plateau ages, scatter the most in their Ca/K value and expose the largest error bars. This is a hint to continued or repeated recrystallization. On the other hand, the fibrous sample BDG-37 from the Berchtesgaden mine, the fibrous sample ALT-53D from the Altaussee mine, and the foliated, well-recrystallized sample DÜ-3B from the Bad Dürrnberg mine show similar Ca/K (^37^Ar/^39^Ar) values, but also large differences in age. Their geochemical composition is roughly the same but going into more detail, even more nuances of chemical differences can be recognized (Supplementary Information 3). However, a systematical analysis would need more data and thus goes beyond the aim of the present study. Most measurements of polyhalite produced disturbed age patterns. The overdispersed data do not define an age, as demanded for an isochron age or a plateau age (Supplementary Information 2). The extent to which the ^37^Ar/^39^Ar represents different stages of growth is uncertain. In any way, the measured ages must be seen as minimum and maximum ages only.

The measurements face yet another difficulty. The patterns often started with a sudden release of argon, which showed an age like that at the end of the measurement. In between, younger ages appeared. Similar patterns were observed earlier during in vacuo experiments of micas (McDougall and Harrison 1999). A differential release of Ar isotopes could be an explanation for these younger ages (Villa 2021). In case of the present study, the younger ages in between could be the result of a breakdown of polyhalite [K_2_Ca_2_Mg(SO_4_)_4_·2H_2_O] to vapor, anhydrite and two langbeinite-type phases (langbeinite [K_2_Mg_2_(SO_4_)_3_], Xu et al. 2017). It is likely that vacancies form during the sudden expulsion of water molecules. A differential release of Ar isotopes based on their atomic masses would shift the age towards younger ages.

No ages of the depositional age at ca. 250 Ma were measured. Only very blurred ages younger than 236 Ma were measured. The Alpine orogeny lasted from upper Jurassic to middle Cretaceous. During this overprint ca. 200 °C were reached. The overprint could thus potentially result from (1) Ar loss, or (2) recovery processes enhanced by fluids and grain size increase. A natural polyhalite was observed to decompose already at 507°K = 233 °C (Xu et al. 2017). This temperature is close to the measured temperatures reached in the salt deposits at ca. 200 °C (Leitner et al. 2014, 2020). The closure temperature of polyhalite might coincide with the decomposition temperature but might be also lower. Unfortunately, Richards et al (2021) determined the closure temperature of langbeinite-type phases, and the closure temperature of polyhalite remains unknown. Moreover, it is known that muscovite and biotite can grow above and below their closure temperature, so consequently, Ar loss by diffusion cannot be totally excluded. However, diffusion is a much slower process than recrystallization by circulating fluids (Villa and Hanchar 2017). The term *hygrochronometer* was proposed to address datable minerals that record circulating fluids (e.g., Bosse and Villa 2019). There are numerous hints from microstructures for the recrystallization of polyhalite from the Alpine deposits. Under the microscope many recovery processes were identified, such as subgrain rotation recrystallisation, twinning, and grain boundary migration (Figs. 3, 4, 5). Villa (2021) discussed the recrystallization of micas on the scale of ≤ 20 µm that can lead to slightly different phases or compositions, and, of course, age. Only small amounts of fluid are sufficient for recrystallization, and fluids can be remobilized many times from grain boundaries during differential tectonic stress. This is especially the case for highly water-soluble materials (Urai et al. 2008; Desbois et al. 2012). Even a complete recrystallization is possible as seen from moving grain boundaries across inclusions during in situ observations (Schenk and Urai 2005). Polyhalite is a highly water-soluble mineral. Therefore, the mixture of ages seems to relate most probably to (2) recovery processes.

### The age dating results in the context of the local geology

The new dating results should be seen in complement to previously measured age data (Leitner et al. 2013, 2014, 2020). The earlier results dated obviously foliated structures. During this study samples of macroscopically syn-depositional structures and primary, non-recrystallized looking fabrics were chosen. However, it turned out that what looked undisturbed at first glance was no longer pristine when examined in sufficient detail.

Ages around ~ 235 Ma occur in many samples as the oldest age steps. ALT-28 was the only sample, which gave consistent plateau ages in repeated experiments at around ~ 235. These plateau results should be seen with caution however, regarding the broad scatter of ^37^Ar/^39^Ar (= Ca/K) ratios. Types 1 and 2 yielded similar ages of ~ 233–236 Ma and ~ 232–233. Type 4 bedded polyhalite with a blocky fabric yielded age steps of ~ 236 Ma and 233 Ma. These oldest ages represent only minimum ages but are contemporaneous with the ones that defined plateau ages for sample ALT-28 Ma. The age at early Carnian is well known for extensive fluid flow within the western, central and eastern Northern Calcareous Alps. Synthetic ore mineralization affected the early Carnian carbonate platforms (Weber 1997a, b; Henjes-Kunst et al. 2014; Prochaska 2016). Between c. 230 and 240 Ma pelagic deep-water sediments were deposited in the region, which were interpreted to relate to the aborted Meliata rift (Kozur 1991; Mandl and Ondrejičková 1991; Neubauer et al. 2000). This rift could have been a potential heat and fluid source. However, from the status quo of knowledge, a sound interpretation of a massive fluid event at 235 Ma is limited at the moment.

Later deformations of the salt relate to salt diapirism and to the Alpine orogeny. There are hints to Triassic diapirs, which were squeezed and extruded to the surface during the Jurassic (Fernandez et al. 2020). Alpine deformational events in evaporites were dated between ca. 145 Ma and 90 Ma by K-feldspar and white mica associated with temperatures as high as 250 °C (Spötl et al. 1998; Frank and Schlager 2006; Dallmeyer et al. 1998; Bojar et al. 2016).

## Conclusions

Sedimentologic and diagenetic structures were found, which prove a syn-depositional origin of polyhalite in the Eastern Alps. Such structures have never been described before from the Alpine deposits but put them in a row with other polyhalite deposits worldwide.

Nearly all measurements of primary or euhedral to subhedral looking grain shapes of polyhalite produced overdispersed data that do not define an age. The data do not meet the criteria of reproducibility, but nonetheless the data contain some information. Most important, the age data represent at least maximum and minimum ages.

Water-based recrystallization processes can be reactivated from fluids along grain boundaries, and recrystallization can occur on very small scale. Also, other recovery processes enhance the reorganization of the crystal. Recovery processes such as subgrain rotation recrystallization, twinning, grain boundary migration and grain size increase were seen in many examples under the microscope. Because polyhalite is a highly water-soluble mineral, these processes, mainly based on the presence of water, are the suggested reason for the virtual scattered ages of polyhalite.

One cutback of the present study was the insufficient spatial resolution of the studied samples. Optical microscopy revealed many different structures. However, element mapping down to 1 µm or a systematic analysis of Ca, K and Cl by the Ar–Ar method would be helpful to identify homogeneous parts of the material. In future studies, back scattered electron images could be used to testify that the measured grains actually consist of single crystals. Due to its easy recrystallization property, polyhalite potentially can serve to date halite deformational events. However, only extremely careful separation of single crystals or in situ age dating under the microscope will be successful in dating polyhalite. Common-denominator three-isotope correlation diagrams (CDTIC) could be used systematically to diagnose mineral mixtures and identify the Ca/K, Cl/K and ^40^Ar*/K ratios of the mixing end-members (Villa 2016; Villa and Hanchar 2017).

Further investigations should test other undestroyed polyhalite occurrences. More baseline studies on polyhalite are necessary to understand its recrystallization and degassing behavior to properly define its potential as a geochronometer.

### Electronic supplementary material

Below is the link to the electronic supplementary material.

Supplementary file1 (XLSX 17 KB) EMPA measurements on polyhalite

Supplementary file2 (XLSX 3182 KB) Raw Data and Plots of ^40^Ar/^39^Ar age dating

Supplementary file3 (PDF 277 KB) Comments of reviewer Igor M. Villa

## Appendix

Supplementary Information 1 contains EMPA measurements on polyhalite. Supplementary Information 2 provides data and plots of ^40^Ar/^39^Ar age dating. Supplementary Information 3 reports tests on the data by the reviewer Igor M. Villa. All supplementary information can be found in the data repository PANGAEA (http://www.pangaea.de), https://doi.org/10.1594/PANGAEA.942487.
