# Supplementary material for: Dating of polyhalite: a difficult 40Ar/39Ar dating tool of diagenetic to very low-grade metamorphic processes
Source: Int J Earth Sci. 2022 Jul 7;111(6):2037–51. doi: 10.1007/s00531-022-02219-9 (PMC9334404; doi:10.1007/s00531-022-02219-9)
Supplement: Supplementary file 3 — Supplementary file3 (PDF 277 KB) Comments of reviewer Igor M. Villa [file 531_2022_2219_MOESM3_ESM.pdf]

Supplementary information (shortened and slightly modified review of Igor Villa):

For the 37Ar/39Ar ratio, (…) only 7 out of 17 samples approach the production ratio of 37Ar and 39Ar during irradiation (0.543, as calculated from the nuclear cross-sections by Onstott et al., 1995, Geochim Cosmochim Acta, their p. 1823). The mismatch of the measured ratios with the calculated ones is variable between 0.76 and 1.18, with a median of 0.9 - in the uppermost panel 1 of my attachment, I plot the (37Ar/39Ar)(meas/calc), of course only for those samples whose 37Ar decay correction was made correctly. The mismatch with the Onstott calculation is worth an investigation, but what is especially striking is (…) the variation of the measured values (even leaving aside the four lowest and the four highest) (…). This is probably not just typographic errors, but points to a serious analytical, or mineralogical, bias.

(…) Quite simply, the "pure salt" analyses in the text must be the lower limit: if the salt monitor gives a (38Ar/39Ar)K ratio of 0.0117, then the measured ratio (consisting of the K-produced 38Ar, *plus* the atmospheric 38Ar, *plus* the Ca-produced 38Ar, *plus* any Cl-derived 38Ar) cannot be lower! There are several artefacts that can produce an incorrect ratio; the two most obvious ones, in my mind, are an incorrect machine background subtraction, or a sloping baseline on the shoulder of a spread-out 40Ar peak (which would occur if the gas was not properly cleaned (…)), in which case the 39Ar intensity would be too high and the (38Ar/39Ar)K ratio too low (as observed).

There are only 7 out of 17 analyses with a correct correction for the decay of 37Ar (as evidenced by the 37Ar/39Ar ratios). (…) Note carefully that this inaccuracy is not just a curiosity; rare gas systematics (as used by Merrihue 1966, J Geophys Res, for Kr and Xe) recommend the use of common-denominator diagnostic diagrams such as the 37Ar/39Ar vs 40Ar/39Ar correlation, but only if the former ratio is correct.

Both isochrons calculated on ALT-11_09, core and rim (s0699 and s0716) have positive slopes! There is no way Isoplot (the software used to draw the graphs in (…) Supplementary Information 2) could ever calculate an age out of a positive slope. (…) Initially I thought, basing on s0716, that maybe a simple mislabeling had occurred, and replotted the data (panels 2 and 3 of my attachment). What I did first, suspecting that the high MSWD was the effect of mixing different minerals (Villa & Hanchar 2017, American Mineralogist), was plotting the 37Ar/39Ar vs 40Ar/39Ar correlation (middle panel). Three points are marked with open/yellow filling: steps 2, 3 and 7. These steps are a different mineral from steps 4, 5 and 6, since their 37Ar/39Ar fingerprint is different, and must not be regressed together in the isochron (bottom panel). Here comes the next surprise: steps 4, 5 and 6 are resolvably discordant, but still are aligned on a positive isochron slope. Because a positive slope cannot define an age, it is obvious that these three points are once again a mixture, and indeed their 38Ar/39Ar ratios (…) are discordant.

Researchers be warned!

(Christoph Leitner)

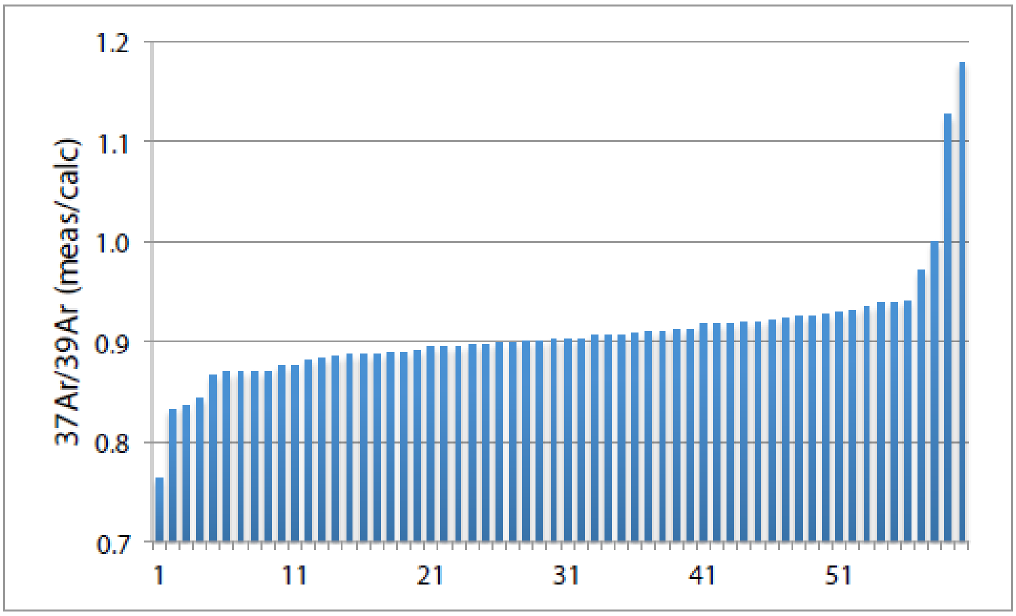

37Ar/39Ar (meas/calc)
1.2
1.1
1.0
0.9
0.8
0.7
1
11
21
31
41
51

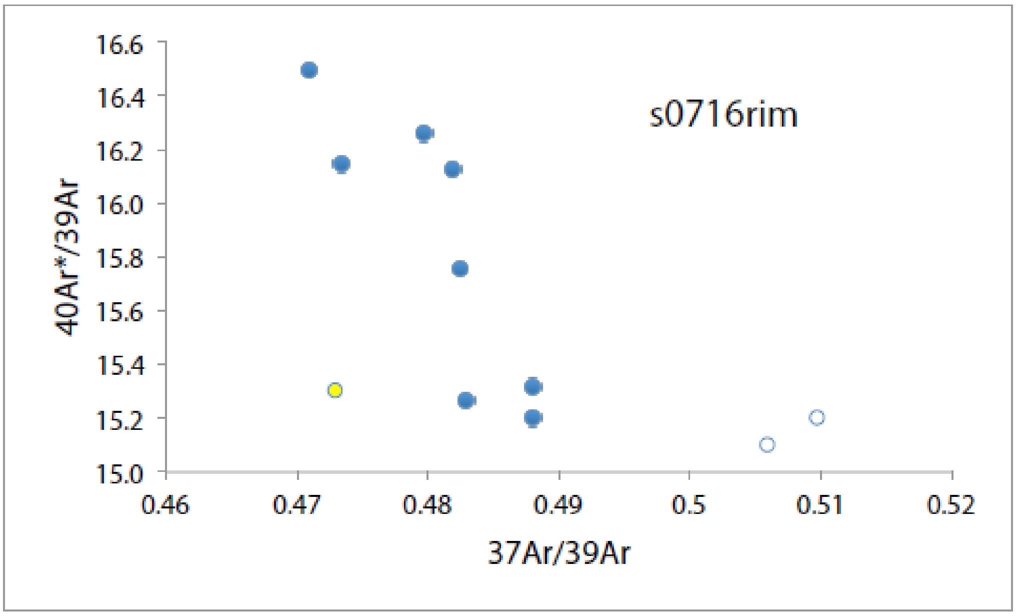

40Ar*/39Ar
37Ar/39Ar
s0716rim
16.6
16.4
16.2
16.0
15.8
15.6
15.4
15.2
15.0
0.46
0.47
0.48
0.49
0.5
0.51
0.52

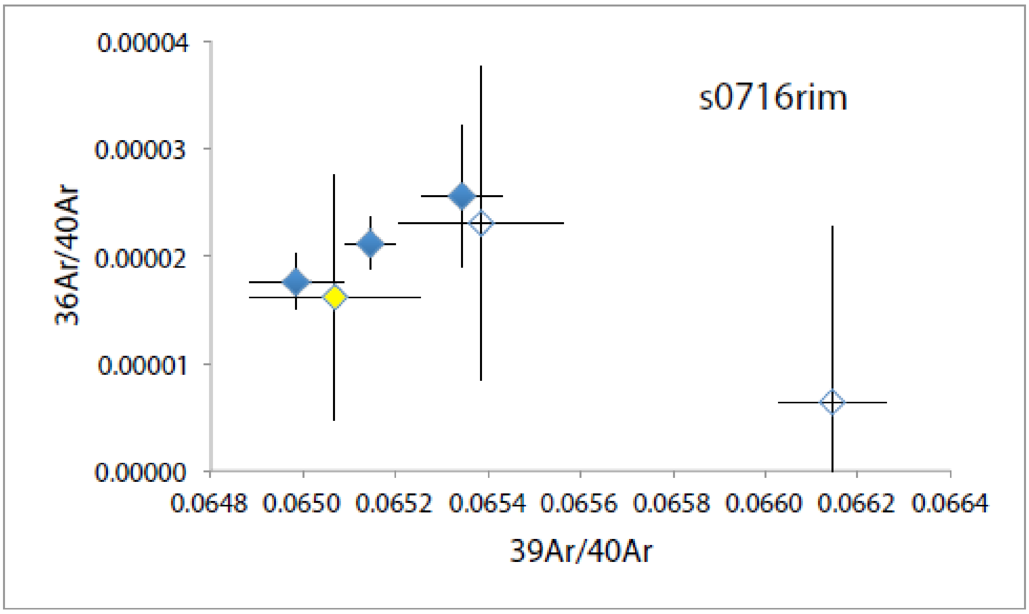

36Ar/40Ar
39Ar/40Ar
s0716rim
0.00004
0.00003
0.00002
0.00001
0.00000
0.0648
0.0650
0.0652
0.0654
0.0656
0.0658
0.0660
0.0662
0.0664
